# A retrospective study of the role of hypercapnia in patients with acromegaly

**DOI:** 10.1186/s12890-023-02488-3

**Published:** 2023-05-27

**Authors:** Junwei Guo, Wenhao Cao, Jinmei Luo, Rong Huang, Yi Xiao

**Affiliations:** 1 Department of Respiratory and Critical Care Medicine, Peking Union Medical College Hospital, Chinese Academy of Medical Sciences & Peking Union Medical College, No. 1 Shuaifuyuan Street, Dongcheng District, Beijing, 100730 China; 2 Department of Respiratory and Critical Care Medicine, The Central Hospital of Wuhan, Tongji Medical College, Huazhong University of Science and Technology, Wuhan, China

**Keywords:** Hypercapnia, Acromegaly, Obstructive sleep apnea, Biochemical remission

## Abstract

**Background:**

Acromegaly is a multisystemic disease characterized by an excessive release of growth hormone (GH) and insulin-like growth factor-1. Obstructive sleep apnea (OSA) is a common consequence of acromegaly, and hypercapnia is frequently observed in patients with acromegaly, OSA, and obesity. However, the effects of hypercapnia on acromegaly remain unknown. This study was designed to investigate whether there are differences in clinical symptoms, sleep variables, and biochemical remission after surgery for acromegaly in patients with OSA with or without hypercapnia.

**Methods:**

A retrospective analysis was conducted involving patients with acromegaly and OSA. The pharmacotherapy history for acromegaly before surgery, anthropometric measures, blood gas, sleep monitoring data, and biochemical assays of hypercapnic and eucapnic individuals were collected 1–2 weeks before surgery. Univariate and multivariate logistic regression analyses were performed to determine the risk factors for failed postoperative biochemical remission.

**Results:**

In this study, 94 patients with OSA and acromegaly were included. Among them, 25 (26.6%) had hypercapnia. The hypercapnic group had higher body mass index (92% vs. 62.3%; *p* = 0.005) and poorer nocturnal hypoxemia index. No serological differences were found between the two groups. According to the post-surgery GH level, 52 patients (55.3%) reached biochemical remission. Univariate logistic regression analysis revealed that diabetes mellitus (odds ratio [OR], 2.59; 95% confidence interval [CI], 1.02–6.55), instead of hypercapnia (OR, 0.61; 95% CI, 0.24–1.58), was associated with lower remission rates. Patients who received pharmacotherapy for acromegaly before surgery (OR, 0.21; 95% CI, 0.06–0.79) and had higher thyroid-stimulating hormone levels (OR, 0.53; 95% CI, 0.32–0.88) were more likely to have biochemical remission after surgery. Multivariate analysis further showed that only diabetes mellitus (OR, 3.29; 95% CI, 1.15–9.46) and preoperative pharmacotherapy (OR, 0.21; 95% CI, 0.06–0.83) remained significant. Hypercapnia, hormone levels, and sleep indicators had no effect on biochemical remission after surgery.

**Conclusions:**

Single-center evidence shows that hypercapnia alone may not be a risk factor for lower biochemical remission rates. Correcting hypercapnia does not appear to be required before surgery. More evidence is needed to further support this conclusion.

## Background

Acromegaly is a slow-progressing clinical illness that affects more than 13 individuals of 100 000 [1]. It is characterized by excessive secretion of growth hormone (GH) and insulin-like growth factor-1 (IGF-1), which is caused by a GH-secreting pituitary tumor in most cases and pituitary hyperplasia or ectopic GH or GH-releasing hormone secretion in rare cases. Apart from endocrine problems, active acromegaly could further lead to cardiovascular, pulmonary, and metabolic comorbidities [2].

The primary goals of treatment include symptom relief, tumor control, and reversal of the morbidity and mortality [3]. Transsphenoidal selective adenomectomy (TSA) is the first-line treatment with reported biochemical remission rates ranging from 30 to 85% [4]. Other medical treatments include somatostatin analogs (SSAs) and stereotactic radiosurgery. It is estimated that in the United States, compared with the general population, uncontrolled acromegaly resulted in $285,000 additional comorbidity-related costs, 0.9 fewer years of life, 4.2 fewer quality-adjusted life years, and 1.6 more comorbidities across the remaining lifespan [5]. The huge disease burden for patients with acromegaly makes long-term biochemical remission indispensable, which could drastically reduce the mortality risk of acromegaly to an equivalent level to that in the general population [6].

Obstructive sleep apnea (OSA) and respiratory insufficiency are the most frequent respiratory complications observed in patients with acromegaly because of anatomical changes, including the bone and soft tissues of the craniofacial region, respiratory mucosa/cartilages, lung volumes, and rib cage geometry [7]. Hypercapnia could be presented in acromegaly, particularly in overweight cases. The role of chronic hypercapnia has been well studied in chronic obstructive pulmonary disease (COPD) and acute respiratory failure, with controversial conclusions [8, 9]. Some studies highlighted the negative impact of hypercapnia on respiratory and metabolic diseases [10], whereas others claimed that it had no effect on mortality [11]. Although hypercapnia is a major laboratory finding in obesity hypoventilation syndrome (OHS), because of the exclusionary criteria [12], patients with OSA accompanied by acromegaly cannot be directly diagnosed with OHS. So far, little study has been conducted on the effect of hypercapnia in patients with OSA and acromegaly.

In this study, we conducted a retrospective study to evaluate whether there were differences in clinical symptoms, sleep variables, and biochemical remission after surgery in patients with acromegaly with or without hypercapnia. The influence of potential risk factors, such as hypercapnia, on biochemical remission was further assessed.

## Methods

### Study population

**Fig. 1:**
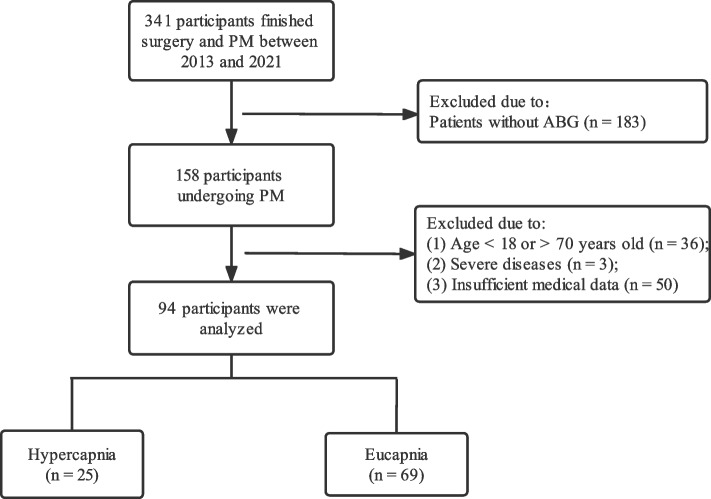
Flow chart of the study population. PM, Portable monitoring; ABG, Atrial blood gas

Patients admitted to the Neurosurgery Department of Peking Union Medical College Hospital (PUMCH) from 2013 to 2021 were enrolled in this study. The inclusion criteria were as follows: (1) patients diagnosed with active acromegaly according to the Endocrine Society Guidelines [13] (elevated IGF-1 levels and unsuppressed GH in the oral glucose tolerance test (OGTT)); (2) those who went through TSA during hospitalization; and (3) those who completed overnight sleep recording and arterial blood gas analysis before surgery. The exclusion criteria were as follows: (1) patients aged < 18 or > 70 years; (2) those who were pregnant or had severe diseases, such as kidney failure, liver failure, or cancer; (3) those with a history of surgery for acromegaly before sleep recording; (4) those receiving long-term domiciliary oxygen therapy or bi-level positive airway pressure use before admission; and (5) those with insufficient medical data. This study was conducted according to the Declaration of Helsinki and was approved by the Ethics Committee of PUMCH. Moreover, obtaining informed consent from the patients was unnecessary because no information regarding privacy was collected. A flowchart for the study population selection and enrollment is presented in Fig. 1.

### Demographic characteristics and sleep recording

Data on the baseline demographic characteristics, including sex, age, weight, height, comorbidities including hypertension and diabetes, use of pharmacotherapy before surgery for acromegaly, disease duration, and current smoking status, were obtained and recorded. A full night portable sleep recording was performed using Embla X100 (Embla, UK) for at least 7 h. Signals, including nasal airflow, pulse oxygen saturation (SpO_2_), sleep positions, and thoracic and abdominal movements, were collected. An experienced sleep laboratory technician reviewed the recording data for analysis according to the criteria listed in the 2017 American Academy of Sleep Medicine [14]. The apnea hypopnea index (AHI) was defined as the average number of apnea and hypopnea events each hour. The diagnosis of OSA was made according to the third edition of the International Classification of Sleep Disorders [15]. OSA severity was classified as follows: mild OSA (15 events/h > AHI ≥ 5 events/h); moderate OSA (30 events/h > AHI ≥ 15 events/h); and severe OSA (AHI ≥ 30 events/h). Nocturnal hypoxemia metrics, such as the oxygen desaturation index (ODI), mean and lowest values of SpO_2_ (LSpO_2_), and the percentage of time spent at SpO_2_ < 90% in total sleep time (T90) during sleep, were also collected.

### Biological measurements

Laboratory tests for acromegaly were performed 1–2 weeks before surgery and were recorded in the electronic medical recording system. Blood routine examination, arterial blood gas, serum lipid, uric acid (UA), fasting blood glucose (FBG), random GH, IGF-1, total cortisol, prolactin, and thyroid function parameters were retrospectively collected. The nadir and random GH within 1 week after surgery were also collected. Arterial blood was drawn when the patients were in the sitting position and breathed room air. An arterial blood gas analyzer (ABL800, Radiometer, Copenhagen, Denmark) was used to analyze the potential of hydrogen, partial pressure of oxygen (PaO_2_), carbon dioxide (PaCO_2_), and bicarbonate. The criterion for postoperative biochemical remission was defined as a random or nadir GH after OGTT < 1 ug/L [16]. Patients with hypercapnia were defined as PaCO_2_ ≥ 45 mmHg, while the rest were considered eucapnic controls. Body mass index (BMI) was calculated as weight (kg) divided by height squared (m^2^); BMI > 25 kg/m^2^ was defined as obesity [17]. The disease duration was calculated as the occurrence of symptoms related to acromegaly to the date of surgery.

### Statistical analysis

All data were analyzed using Statistical Package for the Social Sciences (version 24.0, IBM Corp., Armonk, NY, USA). The normality of the variables was tested using the Kolmogorov–Smirnov test. Categorical variables are described as numbers with percentages. Continuous variables are expressed as means ± standard deviations or medians with interquartile ranges (25%–75%) depending on whether the data were normally distributed. Comparisons between groups were analyzed using the chi-square test, Fisher’s exact test, and unpaired, two-tailed t-test. The non-parametric Mann–Whitney U-test was used when the data were not normally distributed. Logistic regression analysis was used to determine the risk factors for failed postoperative biochemical remission. Covariates with *p*-values < 0.1 after the univariate analysis or supposed to be clinically significant will be reexamined by multivariate analysis. Two-sided *p*-values of less than 0.05 were used to indicate statistical significance.

## Results

### Baseline characters

In this study, 94 patients who fulfilled the inclusion criteria were recruited. The prevalence of hypercapnia was 26.6% (25/94) in the study population. Table 1 shows the basic demographic characteristics and sleep parameters between eucapnic and hypercapnic patients with acromegaly. The hypercapnic group had more patients with obesity (BMI > 25 kg/m^2^) than the eucapnic group (92% vs. 62.3%; *p* = 0.005). No significant differences in age, sex, BMI, and smoking status were observed between the two groups. 17 patients received pharmacotherapy before surgery. The disease duration and use of pharmacotherapy for acromegaly before surgery were not significantly different. As for the sleep recordings, although no significant difference in the AHI was found, the hypercapnic group had a higher proportion of patients with severe OSA (52% vs. 27.5%; *p* = 0.027). Moreover, patients with hypercapnia showed worse nocturnal hypoxemia variables, including the ODI (29.4% vs. 14.4%; *p* = 0.035), LSpO_2_ (79% vs. 85%; *p* = 0.013), and T90 (1.9% vs. 0.4%; *p* = 0.012).

**Table 1:** Basic demographic and sleep parameters of the patients with acromegaly

Variables	Eucapnic acromegaly (*n* = 69)	Hypercapnic acromegaly (*n* = 25)	*P* value
Age, y	46.6 ± 11.4	45.9 ± 12.9	0.836
Male, n (%)	40 (57.8%)	18 (72.0%)	0.139
BMI, kg/m^2^	26.2 (24.1–29.4)	27.1 (26.0–29.7)	0.079
BMI > 25, n (%)	43 (62.3%)	23 (92.0%)	0.005
disease duration, y	6.0 (3.0–10.0)	5.0 (3.0–7.0)	0.763
SSA before surgery	12 (17.4%)	5 (20.0%)	0.768
DM, n (%)	17 (24.6%)	9 (36.0%)	0.277
Hypertension, n (%)	26 (37.7%)	12 (48.0%)	0.368
Current smoking, n (%)	8 (11.6%)	4 (16.0%)	0.572
Supine-AHI, /h	24.7 (11.5–49.5)	41.6 (16.0–53.9)	0.409
AHI, /h	14.6 (8.1–31.2)	37.8 (10.8–57.4)	0.076
AHI > 30, n (%)	19 (27.5%)	13 (52.0%)	0.027
ODI, /h	14.4 (6.1–25.1)	29.4 (9.9–58.2)	0.035
LSpO_2_, %	85.0 (79.5–89.0)	79.0 (66.0–84.0)	0.013
T90, %	0.4 (0.0–3.3)	1.9 (0.3–26.8)	0.012

*BMI* Body mass index, *SSA* Somatostatin analogue, *DM* Diabetes mellitus, *AHI* Apnea hypopnea index, *ODI* Oxygen desaturation index, *LSpO*_*2*_ Lowest pulse oxygen saturation, *T90* Time spent with SpO2 < 90%

### Laboratory tests

Table 2 shows the laboratory findings during the perioperative period between the two groups. Among the patients included in this study, 52 (55.3%) reached postoperative biochemical remission. We found no differences in the metabolic profiles, such as FBG, UA, total cholesterol, triglyceride, and lipoproteins. Hormone levels, such as thyroid function parameters, total cortisol, and prolactin, were maintained. No statistically significant differences in disease condition, such as preoperative random fasting GH and IGF-1 levels, and the proportion of postoperative nadir GH in the OGTT > 1 were observed.

**Table 2:** Laboratory findings of the patients with acromegaly

Variables	Eucapnic acromegaly (*n* = 69)	Hypercapnic acromegaly (*n* = 25)	*P* value
WBC, × 10^9^/L	5.0 (4.1–6.7)	4.8 (4.2–6.2)	0.387
TC, mmol/L	4.2 ± 1.0	4.0 ± 0.6	0.192
TG, mmol/L	1.3 (1.0–1.7)	1.1 (0.8–1.6)	0.614
HDL, mmol/L	1.1 ± 0.3	1.0 ± 0.2	0.167
LDL-c, mmol/L	2.5 ± 0.7	2.3 ± 0.5	0.238
UA, μmol/L	292.8 ± 86.9	297.2 ± 60.2	0.810
FBG, mmol/L	5.8 (5.3–6.4)	5.4 (4.9–5.9)	0.098
Preoperative IGF-1, ng/mL	778.4 ± 314.4	869.9 ± 235.8	0.123
Preoperative GH, μg/L	9.5 (5.8–21.8)	15.4 (8.4–52.0)	0.183
Postoperative GH, μg/L	0.96 (0.46–2.0)	0.61 (0.3–1.5)	0.116
Postoperative GH > 1, n (%)	33 (47.8%)	9 (36%)	0.308
TSH, μIU/mL	1.0 (0.5–1.9)	1.2 (0.6–2.3)	0.399
FT3, pg/ml	3.3 (2.7–3.6)	3.2 (3.0–3.6)	0.765
FT4, ng/ml	1.2 (1.1–1.3)	1.2 (1.1–1.4)	0.864
Total cortisol, mg/dl	10.2 (7.4–16.3)	10.6 (7.5–13.4)	0.854
Prolactin, ng/ml	10.9 (6.2–16.6)	11.7 (9.9–16.7)	0.088

*WBC* White blood cell, *TC* Total cholesterol, *TG* Triglyceride, *HDL* High-density lipoprotein cholesterol, *LDL-c* Low-density lipoprotein cholesterol, *UA* Uric acid, *FBG* Fasting blood glucose, *IGF-1* Insulin-like growth factor-1, *GH* Growth hormone, *TSH* Thyroid stimulating hormone, *FT3* Free triiodothyronine, *FT4* Free thyroxine

### Potential risk factors for low biochemical remission

We further performed univariate and multivariate logistic regression analyses to explore the potential factors for unsuccessful postoperative biochemical remission. Demographic characteristics, pharmacotherapy for acromegaly, sleep indicators, and biological measurements along with hypercapnia were examined. The results are demonstrated in Table 3. In the univariate analysis, medical history of diabetes mellitus (odds ratio [OR], 2.59; 95% confidence interval [CI], 1.02–6.55), instead of hypercapnia (OR, 0.61; 95% CI, 0.24–1.58), statistically increased the likelihood of procedure failure. In contrast, the preoperative use of pharmacotherapy for acromegaly (OR, 0.21; 95% CI, 0.06–0.79) and higher thyroid-stimulating hormone levels (OR, 0.53; 95% CI, 0.32–0.88) were associated with a higher probability of achieving postoperative biochemical remission. In the multivariate analysis, the prognostic value of diabetes and medical therapy remained significant (OR, 3.29 and 0.21; 95% CI, 1.15–9.46 and 0.06–0.83, respectively). However, hypercapnia, as well as hormone levels and sleep indicators, could not significantly influence postoperative biochemical remission.

**Table 3:** Logistic regression analysis of factors associated with postoperative biochemical remission in patients with acromegaly

Variables	Univariate analysis		Multivariate analysis	
	OR (95% CI)	*P* value	OR (95% CI)	*P* value
Hypercapnia	0.61 (0.24–1.58)	0.31	-	-
BMI > 25	0.90 (0.37–2.20)	0.824	-	-
Age > 40	1.13 (0.48–2.61)	0.784	-	-
Duration of symptoms	1.06 (0.98–1.14)	0.132	-	-
Male	0.45 (0.19–1.05)	0.063	-	-
DM	2.59 (1.02–6.55)	0.045	3.29 (1.15–9.46)	0.027
Hypertension	0.84 (0.37–1.93)	0.679	-	-
Current smoking	1.88 (0.55–6.42)	0.314	-	-
LSpO_2_	1.04 (0.99–1.09)	0.088	-	-
ODI	0.99 (0.97–1.01)	0.137	-	-
T90	0.97 (0.93–1.00)	0.059	-	-
AHI > 30/h	0.43 (0.17–1.05)	0.063	-	-
Preoperative pharmacotherapy	0.21 (0.06–0.79)	0.020	0.21 (0.06–0.83)	0.034
TSH	0.53 (0.32–0.88)	0.013	-	-
Total cortisol	0.93 (0.86–1.00)	0.057	-	-
Prolactin	1.02 (0.99–1.04)	0.182	-	-

*BMI* Body mass index, *DM* Diabetes mellitus, *LSpO*_*2*_ Lowest pulse oxygen saturation, *ODI* Oxygen desaturation index, *T90* Time spent with SpO2 < 90%, *AHI* Apnea hypopnea index, *TSH* Thyroid stimulating hormone, *OR* Odds ratio, *CI* Confidence interval

## Discussion

In this study, we compared the effects of hypercapnia with those of eucapnia on patients with OSA and acromegaly. The hypercapnic group had higher BMI and poorer nocturnal hypoxemia parameters than the eucapnic group. A further logistic regression analysis found that diabetes, instead of hypercapnia, was a risk factor for a lower probability of achieving postoperative biochemical remission, whereas preoperative medical treatment was associated with long-term biochemical remission. The result remained significant in the multivariate analysis.

Chronic daytime hypercapnia is caused by decreased minute ventilation/global hypoventilation, increased dead space, or increased carbon dioxide (CO_2_) production. Diseases related to the nervous system, respiratory muscles, and upper airway or lungs can contribute to hypercapnia. Respiratory acidosis has controversial clinical effects attributed to the overproduction of hydrogen ions (H^+^). While it could lead to the favorable effects, such as improvement in gas exchange and protection of ventricular function, excessive H^+^ could reduce diaphragmatic contractility, which is the main damage to the respiratory system [18, 19]. Cardiovascular instability, hypotension, and decline in neurocognitive function are other end-organ side effects [20, 21].

Hypercapnia could result from acromegalic complications, such as OSA and obesity. However, studies examining the mechanism and influence of hypercapnia on acromegaly are scarce. Several studies focused on the effects of chronic metabolic acidosis on the GH/IGF-1 endocrine axis. This situation could be reflected by growth retardation in children suffering from chronic acidosis and reduced bone mass in adults. Animal experiments have found that the expression of GH and IGF-1 receptors is suppressed under acidic conditions at both the mRNA and protein levels, whereas the expression of IGF-binding proteins 2 and 4 is enhanced, which could inhibit IGF-1 activity [22, 23]. Human trials revealed that chronic metabolic acidosis reduces the serum concentration of IGF-1 and is associated with a resistance to the hepatocellular action of GH [24]. These studies showed the interference of acidosis with the GH/IGF-1 endocrine axis. The same acidic condition induced by hypercapnia may have similar results, and thus, it may affect some aspects of acromegaly, including biochemical remission.

Our results showed that the hypercapnic group had a higher proportion of obesity and worse nocturnal hypoxemia indicators than the eucapnic group. The possible explanations to these are listed as follows: (1) hypercapnia could be an indicator of OSA severity. Kaw et al. compared hypercapnic and eucapnic patients with OSA and concluded that daytime hypercapnia was associated with the severity of OSA, higher BMI levels, and degree of restrictive chest wall mechanics [25]. Furthermore, it has been proven that daytime hypercapnia and nocturnal hypoxia are independent predictors of CPAP failure in patients with OSA and COPD [26]. (2) As previously stated, continuous hypercapnia reduces diaphragmatic contractility. In OSA, this effect could be exacerbated by nocturnal hypercapnia. Severe acidosis may reduce the central respiratory drive, resulting in a depressed level of consciousness (known as CO_2_ narcosis) and hypoxemia. (3) Apart from OSA, hypercapnia could be observed in OHS and COPD. OHS is characterized by high BMI, hypercapnia, and hypoxemia [27]. COPD and OSA could be presented together, which is known as the overlap syndrome [28]. Because of the lack of pulmonary function data, the validation of these complications is limited.

There seemed to be little difference in the metabolic and hormonal profiles between the two groups. The negative results may be due to the following reasons: (1) the overall severity of PaCO_2_ was mild to moderate in our clinical samples. The average pH level in the aforementioned human trials reached 7.31, while the pH of our samples was 7.39 ± 0.03. Mild acidosis may have little impact on the GH/IGF-1 endocrine axis. (2) The blood gas analysis was performed a week within the procedure. Only the hypercapnia status rather than its duration could be confirmed. Most pieces of previous evidence were based on the long-term effects of metabolic acidosis. The exact degree and duration of hypercapnia that significantly change the secretion of GH/IGF-1 are unknown. A prospective study involving more severe cases with definite hypercapnia duration is needed for the comprehensive assessment.

To achieve biochemical remission, identifying patients who might benefit from primary medical therapy or require multimodality treatment besides surgery is necessary. Numerous studies have been conducted to determine proper prognostic factors for biochemical remission. Most studies focused on the demographic characteristics and preoperative biochemical and imaging parameters. Though the criterion for postoperative biochemical remission has changed over these years [3, 16], a general consensus of predictors has been reached, which includes cavernous sinus invasion (CSI) by imaging [29–32], larger tumor size [33–35], and higher GH levels [36–38]. Other promising predictive markers include younger age [37, 39], female [30], higher IGF-1 [32, 36], and Knosp grades [34, 39, 40]. A multivariate logistic regression model [31] has been developed based on these parameters. The area under the receiver operating characteristic curve (AUC) is 0.933, whereas the AUC of the model that consists of tumor diameter and CSI only is 0.800 (*p* = 0.02).

Other than reviewing the conclusions of previous studies, our primary goal was to determine whether hypercapnia could be a risk factor for a lower probability of achieving biochemical remission. The results showed that hypercapnia could not significantly influence the postoperative biochemical remission rate. A few details may explain this: (1) acromegaly is a multisystem disorder and hypercapnia alone might exert bilateral influence on endocrine secretion as mentioned in the mechanism above. (2) The severity and duration of hypercapnia may play a more important role in the course of the disease. The negative result may be because of the selection bias of mild hypercapnia in our participants. (3) The small number of participants in this study may not be fully representative of the real situation.

Despite the negative finding, the results suggested that patients with a medical history of diabetes and the use of pharmacotherapy before surgery should have prognostic values. Diabetes and insulin resistance (IR) are responsible for several side effects in patients with acromegaly. Improving insulin sensitivity is a major goal of treatment. Several studies have confirmed that TSA can normalize GH-induced glycol-metabolism disorder and insulin sensitivity [41]. Diabetes could be viewed as a result of GH overproduction, which is a proven marker of biochemical remission. The coexistence of diabetes could alter GH and IGF-1 levels and influence the OGTT result and the clinical judgment of biochemical remission [42]. Furthermore, our previous study has proven that IR is a significant risk factor for cardiovascular disease in patients with acromegaly and OSA [43]. Controlling hyperglycemia throughout the entire course of acromegaly seems plausible.

The use of SSA before surgery could improve the biochemical remission rate, which was proven by our study result. Although the Endocrine Society clinical practice guidelines in 2014 recommended against the routine use of preoperative SSA therapy to improve biochemical control after surgery [13], some studies reported higher surgical control rates with the pretreatment of SSA [44, 45]. Previous data from our center also revealed that prolonged preoperative treatment of acromegaly with SSA (> 6 months) may improve surgical outcomes in patients with invasive pituitary macroadenoma [46]. The use of SSA could alleviate acromegaly symptoms, induce clinically relevant tumor shrinkage, and lower surgical risk by decreasing arterial stiffness; reducing soft tissue swelling, particularly in the upper airways; and inducing better blood pressure control [47]. Despite all these potential advantages, there is still limited data concerning perioperative morbidity and postoperative biochemical outcomes. Currently, the preoperative use of SSA is recommended only in patients with severe cardiac and respiratory complications. More investigation and investment in large randomized long-term clinical trials are needed to define the precise role and duration of preoperative SSA in patients with acromegaly.

It is worth noting that although metrics related to OSA were not significantly associated with the risk of biochemical remission failure, there were protective trends toward significance in some variables, including LSpO_2_ (OR, 1.04; 95% CI, 0.99–1.09; *p* = 0.088), T90 (OR, 0.97; 95% CI, 0.93–1.00; *p* = 0.059), and AHI > 30/h (OR, 0.43; 95% CI, 0.17–1.05; *p* = 0.063) in the univariate analysis. So far, few studies have focused on the influence of the coexistence and severity of OSA on postoperative biochemical remission in patients with acromegaly. It is plausible that OSA, characterized by intermittent hypoxemia and sleep fragmentation, may bring worse outcomes to acromegaly similar to other metabolic complications [48]. However, our results showed the potential protective role of OSA. Because of the relatively small study sample, more participants are needed to further corroborate the significance of the results of this study.

This study has several limitations. First, considering the small sample size and the retrospective nature of this single-center study, the strength of this study is limited. Second, the diagnosis of OSA was based on the result of a portable overnight sleep recording, which contains less information compared to polysomnography and due to this, the severity of OSA might be underestimated. Third, pulmonary function was not evaluated in this study, making it difficult to distinguish the reason for hypercapnia. As mentioned above, patients with chronic respiratory diseases like COPD and OHS often present with hypercapnia. Heterogeneities in the hypercapnic group might be overlooked. Fourth, because of the short-term follow-up of this study, the likelihood of biochemical remission might be misestimated. A large sample size with a randomized study design and long-term follow-up is needed. Different conditions including OHS, COPD or other comorbidities should also be examined. A complete and standard evaluation procedure including polysomnography, pituitary magnetic resonance imaging and blood gas analysis across perioperative period and follow-up should be performed in the future. However, despite these shortcomings, to the best of our knowledge, this is the first study that examined the role of hypercapnia in the clinical symptoms, disease severity, and prognostic value of acromegaly in patients with OSA.

## Conclusions

Patients with acromegaly and hypercapnia are characterized by higher BMI and worse sleep indicators. Diabetes mellitus, instead of hypercapnia, might be a predictor of low probability of achieving postoperative biochemical remission, whereas the preoperative use of SSA therapy may improve the biochemical remission rate. Correcting hypercapnia before surgery seems unnecessary. More attention should be paid to the management of the cause of hypercapnia. Studies are needed to further support the conclusion and determine the potential role of OSA in biochemical remission.

## Data Availability

The datasets used and/or analyzed during the current study are available from the corresponding author on reasonable request.

## Abbreviations

AHI: Apnea hypopnea index
AUC: Receiver operating characteristic curve
BMI: Body mass index
CI: Confidence interval
CO_2_: Carbon dioxide
COPD: Chronic obstructive pulmonary disease
CSI: Cavernous sinus invasion
FBG: Fasting blood glucose
GH: Growth hormone
IGF-1: Insulin-like growth factor-1
IR: Insulin resistance
LSpO_2_: Mean and lowest values of SpO_2_
ODI: Oxygen desaturation index
OGTT: Oral glucose tolerance test
OHS: Obesity hypoventilation syndrome
OR: Odds ratio
OSA: Obstructive sleep apnea
SpO_2_: Pulse oxygen saturation
SSAs: Somatostatin analogs
T90: Percentage of time spent at SpO_2_ < 90% in total sleep time
TSA: Transsphenoidal selective adenomectomy
TSH: Thyroid stimulating hormone
UA: Uric acid
